# Simultaneous Determination of Chrysin and Tectochrysin from *Alpinia oxyphylla* Fruits by UPLC-MS/MS and Its Application to a Comparative Pharmacokinetic Study in Normal and Dementia Rats

**DOI:** 10.3390/molecules23071702

**Published:** 2018-07-12

**Authors:** Xu Zhao, Xin Su, Chunmei Liu, Ying Jia

**Affiliations:** 1 Faculty of Functional Food and Wine, Shenyang Pharmaceutical University, Wenhua Road 103, Shenyang 110016, China; zhaoxu_1010@163.com; 2 Faculty of Traditional Chinese Materia Medica, Shenyang Pharmaceutical University, Wenhua Road 103, Shenyang 110016, China; 15642828518@163.com (X.S.); liuchunmeicjt@163.com (C.L.)

**Keywords:** *Alpinia oxyphylla*, flavonoid, pharmacokinetics, dementia

## Abstract

A simple and rapid ultra-performance liquid chromatography–tandem mass spectrometry (UPLC–MS/MS) coupled with a one-step liquid-liquid extraction method has been developed and validated for the simultaneous determination of two flavonoids (chrysin, tectochrysin) from *Alpinia oxyphylla* fruits extract in rat plasma. Plasma samples were extracted with diethyl ether and separated on an ACQUITY UPLC BEH C_18_ column (100 mm × 2.1 mm, 1.7 μm) using gradient elution consisting of 0.1% formic acid in water (A) and methanol (B). The multiple reaction monitoring (MRM) mode with electrospray ionization in the positive ion mode was used for detection. The linear range was 0.1 ng/mL to 50 ng/mL for chrysin and tectochrysin, respectively. The accuracy (relative error, RE%) ranged from −8.8% to 7.5% and the intra-day and inter-day precision were within 15% and had a mean extraction recovery rate of 80.3% to 86.7%. The validated method was applied to a comparative pharmacokinetic study after oral administration of *Alpinia oxyphylla* fruit ethanol extract between normal rats and dementia rats. The area under the curve (AUC) and peak plasma concentration (*C*_max_) of the two constituents were remarkably increased in dementia rats than in normal rats.

## 1. Introduction

*Alpinia oxyphylla* Miq. (Yizhi in Chinese), which is a well-known health food and traditional Chinese medicine (TCM), is widely distributed in Southern China. It is traditionally applied to treat intestinal and urethral disorders [1]. Modern pharmacological studies have indicated that the fruits of *Alpinia oxyphylla* possess significant pharmacological roles and the underlying mechanisms have been explored by our lab [2,3,4,5] as well as other teams [6,7,8,9,10]. Alzheimer’s disease (AD) is an age-related neurodegenerative disorder, which disrupts various functions of the brain like intelligence, memory, and learning ability. Amyloid-beta (Aβ) is one of the underlying mechanisms of AD [11]. Both overproduction or lack of clearance of Aβ are involved in the neurodegeneration associated with AD [12,13]. Several studies showed that intra-cerebroventricular (i.c.v.) injection of the Aβ_1–42_ peptides could imitate some signs of AD including memory impairment, oxidative stress, and neuro-inflammation, which is an accepted dementia modeling method [2,3,4,14]. In the previous studies, we have demonstrated the neuroprotective activity effects of chloroform and n-butanol extracts from the fruits of *A. oxyphylla* in an Aβ_1–42_-induced AD mouse model. *A. oxyphylla* improved learning and memory deficits by modulating the activation of microglia and the degeneration of neuronal acidophilia, which attenuates oxidative stress and reinforces cholinergic functions [2,3]. The total flavonoids of *A. oxyphylla* exerted antidepressant effects by targeting TrkB receptor-mediated pERK/pCREB/BDNF signal systems [5].

Flavonoids are the main active constituents of *A.* *oxyphylla* fruits, which have been found to exert various pharmacological activities [15,16]. Total and monomeric flavonoid compounds have been found to possess neuroprotective, antioxidant, anti-inflammatory, and anti-apoptosis properties [5,17,18]. These active constituents make a great contribution to the neuroprotective effects for *A. oxyphylla* in vivo. Therefore, it is crucial to decipher their pharmacokinetic (PK) profiles to better elucidate the preclinical characteristics as well as the underlying mechanisms for treatment of a neurodegenerative disorder.

Flavonoids in *A. oxyphylla* showed low levels in plasma because they mainly formed mono-glucuronide metabolites [19] so it is difficult to determine free flavonoids in plasma. As a result, there are only few analytical methods reporting on the active constituents from *A. oxyphylla* in bio-samples [19,20]. No paper has reported on PK or other preclinical study in dementia model rats. In the present study, by improving previous extraction methods, a simple and rapid UPLC–MS/MS coupled with a one-step liquid-liquid extraction method for simultaneous quantitation of chrysin and tectochrysin in rat plasma has been established and validated. The pharmacokinetic profiles after oral administration of ethanol extract of *A. oxyphylla* fruits were investigated. We have offered a promising approach for the in vivo analysis of active constituents and a foundation for the preclinical study of *Alpinia oxyphylla*.

## 2. Results and Discussion

### 2.1. The Morris Water Maze Test

**Figure 1:**
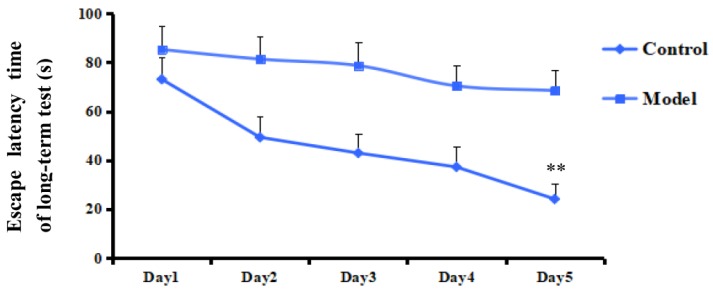
The escape latency time of a long-term test on five days in the Morris water maze test. Data represent means ± S.E.M. (*n* = 6, ** *p* < 0.01 compared with the Control group).

**Figure 2:**
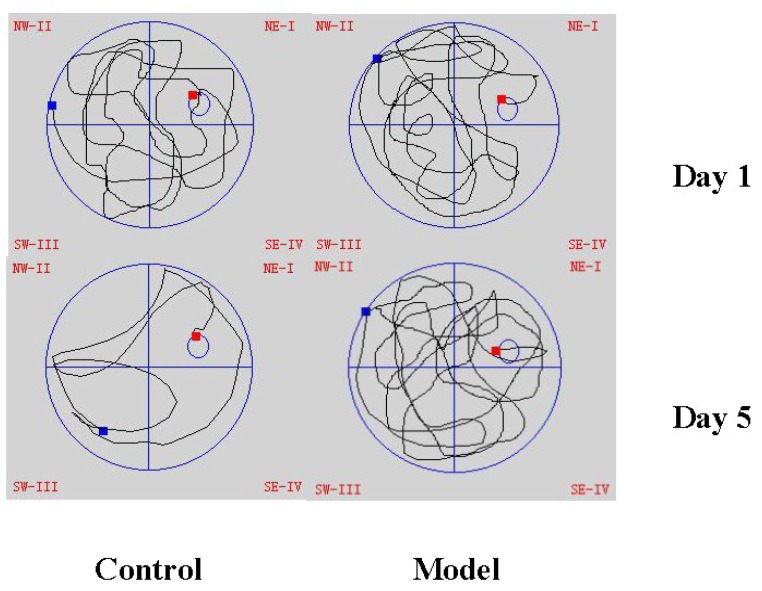
The search strategy of the rats on the first and fifth day in the Morris water maze test (*n* = 6, blue spot: the initial location of the rats, red spot: the final location of the rats).

**Figure 3:**
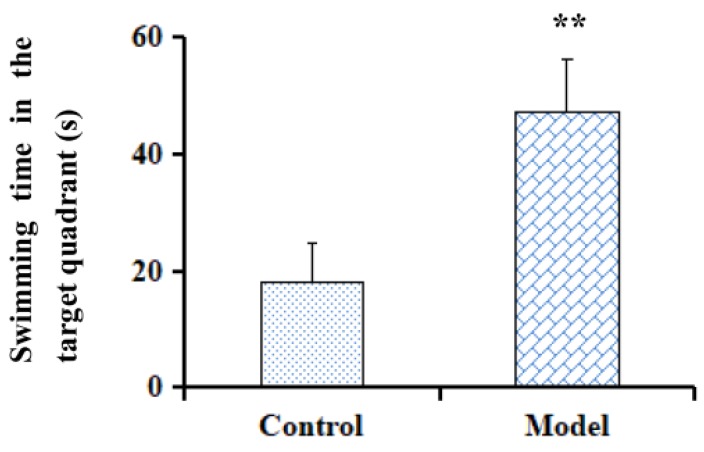
The swimming time in the target quadrant in trial sessions of the Morris water maze test. Data represent means ± S.E.M. (*n* = 6, ** *p* < 0.01 compared with the Control group).

The Morris water maze test is a classic method for evaluating memory and spatial learning ability. The performance of the tested objects were evaluated by parameters including the escape latency time to find a platform and swimming time in the target quadrant. As a result, rats in the model group showed a significant delay on the escape latency time compared with the control group on the fifth day of the training (*p* < 0.01, Figure 1). The search strategies of the rats on Day 1 and Day 5 were shown in Figure 2. Moreover, on the probe testing day (Day 6), rats treated with Aβ_1–42_ spent less swimming time searching for the platform in the target quadrant after the platform was removed (*p* < 0.01, Figure 3). The results indicated that rats treated by Aβ_1–42_ had spatial learning and memory damage in the brain, which led to dementia.

### 2.2. Method Validation

#### 2.2.1. Selectivity

**Figure 4:**
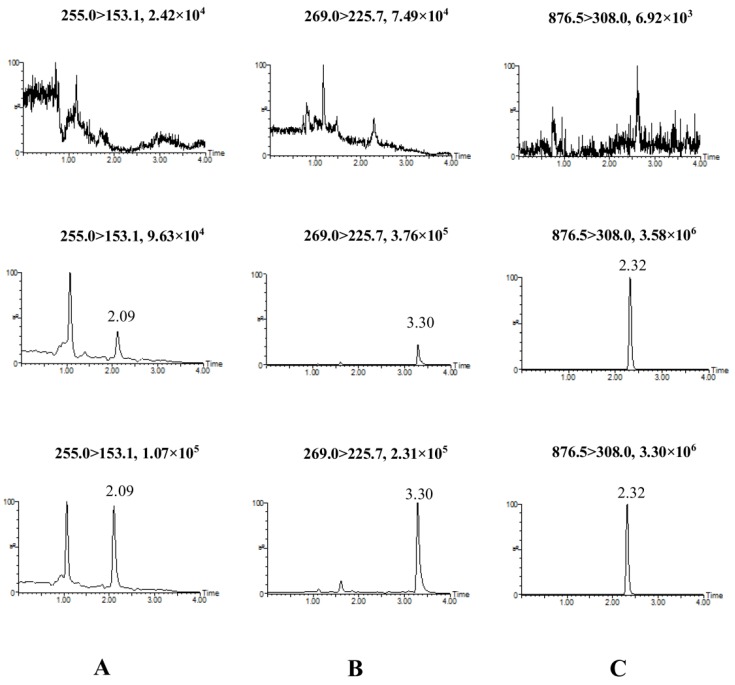
MRM chromatograms obtained from blank rat plasma, blank rat plasma spiked with analytes (0.1 ng/mL, LLOQ) and the IS, and rat plasma 0.25 h after oral administration of *Alpinia oxyphylla* extract. Chromatograms of chrysin (**A**); tectochrysin (**B**); and IS (**C**).

With regard to typical multiple reaction monitoring (MRM) chromatograms of blank plasma, blank plasma spiked with two analytes at a lower limit of quantification (LLOQ, 0.1 ng/mL) and the internal standard (IS). A normal group rat plasma sample 0.25 h after administration of extract of *Alpinia oxyphylla* fruits were shown in Figure 4. There was no endogenous interference being detected at retention times of chrysin (2.09 min), tectochrysin (3.30 min), and paclitaxel (2.32 min, IS), which proves satisfactory selectivity of the assay.

#### 2.2.2. Calibration and LLOQ

The calibration curves showed good linearity over the concentration range in rat plasma. A typical linear regression equation of the calibration curves were *y* = 0.05791*x* − 0.06715 (*r* = 0.9945), *y* = 0.02034*x* + 0.01833 (*r* = 0.9943) with the LLOQ of 0.1, 0.1 ng/mL for chrysin and tectochrysin, respectively.

#### 2.2.3. Accuracy and Precision

The accuracy of intra-day and inter-day precision data of the assays were shown in Table 1. The accuracies were all within ±10.0%. The intra-day and inter-day precisions for all analytes were less than 15%, respectively. All the results of the tested samples were meeting the acceptable criterion (RSD < 15%, RE within ±15%), which indicated that the developed method was accurate and reliable.

#### 2.2.4. Recovery and Matrix Effect

The mean extraction recoveries of the two analytes were all more than 80.3% and the mean recovery of the IS was 82.7%, which indicated that the recovery of analyte was precise and consistent. There was no significant matrix effect affecting the determination of the analytes and IS. The results were presented in Table 1.

#### 2.2.5. Stability

Stability studies of the samples were run at three quality control (QC) levels. The results were shown in Table 2, which demonstrated that all the samples were stable at room temperature for 12 h, at −20 °C for 30 days after three freeze and thaw cycles, and at 4 °C in an auto-sampler for 12 h after being prepared.

### 2.3. Pharmacokinetic Study

**Figure 5:**
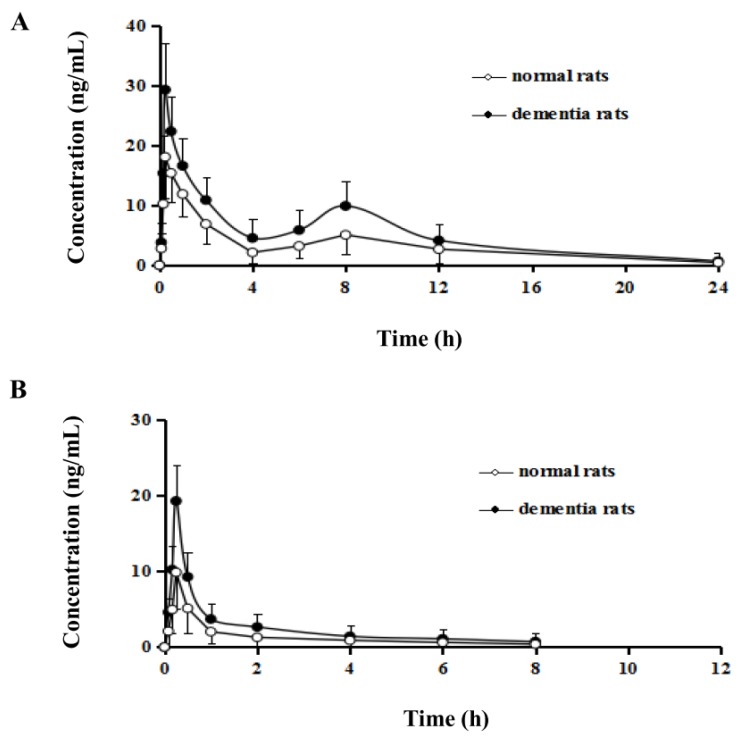
The mean plasma concentration versus time profiles of chrysin (**A**) and tectochrysin (**B**) in rat plasma after oral administration of *Alpinia oxyphylla* fruit extract (*n* = 6).

The utility of the UPLC-MS/MS method for the quantitation of two flavonoids in rat plasma was demonstrated after drug administration. The mean plasma concentration versus time profiles of chrysin and tectochrysin were illustrated in Figure 5. The main pharmacokinetic parameters were listed in Table 3.

As shown in Table 3, two flavonoids showed low AUC and *C*_max_ in plasma due to their relative low levels in the crude drug and partly forming mono-glucuronide metabolites in plasma [19]. Chrysin exhibited rapid absorption and a bimodal phenomenon in plasma concentration—time profiles, which was consistent with the previous studies [21,22]. The first peak occurred at about 0.25 h and the second at 8 h due to the glucuronidation [19] and enterohepatic circulation [23]. Moreover, there were statistically remarkable differences on the pharmacokinetic parameters of AUC, *C*_max_, and CL_F of the two analytes between normal and dementia rats. Compared with the normal rats, the *C*_max_, AUC_0–t_, and AUC_0–∞_ in dementia rats were remarkably increased (*p* < 0.01).The CL_F was decreased significantly (*p* < 0.01) after oral administration of extract of *A. oxyphylla* dried fruits, which indicated a better absorption and slower elimination of the test constituents in an Aβ-induced AD model of dementia rats.

The reason for better uptake of the constituents in AD rats may include the following. AD is a progressive and complex neurodegenerative disorder characterized by extracellular deposited amyloid-β peptide and intracellular hyper-phosphorylated and tangled tau-protein [24]. AD pathogenesis also involves vascular abnormalities such as alterations to the neurovascular unit [25] and decreases in cerebral blood flow [26]. Fibrinogen is a plasma glycoprotein that circulates at a high concentration in the blood and is essential for coagulation since it is converted into fibrin in response to injury [27]. Evidence has indicated a key role for fibrinogen and fibrin clot formation in AD pathogenesis [28,29]. Fibrinogen could increase the viscosity of blood [30] and cause poor blood circulation, which might cause a longer residence time of the drugs in the blood.

The other reason is that, in AD patients, the levels of blood hydroxyl radicals increase regularly [31]. Glutathione, which is a major antioxidant, plays a crucial role in the protection of tissues from radical injury [32]. The cost of glutathione needs more precursor glutamine, which is vital for the production of ATP and the main respiratory material of the small intestine [33]. Flavonoids have been reported to be modulators of P-glycoprotein (Pgp), which could bind strongly to a Pgp cytosolic site [34]. The two constituents are probably to be pumped out by Pgp [35] and the decreased glutamine would cause a decline of ATP levels and a reduction in the function of P-glycoprotein, which is combined with the longer residence time of the drugs in a small intestine. Further pharmacokinetic study of the two constituents after intravenous (i.v.) administration should be carried out to elucidate the hypothesis mentioned above. Essentially, the results indicated that the two flavonoids showed a better absorption rate under pathological conditions in rats.

## 3. Materials and Methods

### 3.1. Materials and Reagents

*Alpinia oxyphylla* fruits were obtained from the Shenyang Tongrentang Drug Company (Shenyang, China) and the source of the herbs is the Hainan province. Chrysin, tectochrysin (purity > 99.8%), and paclitaxel (purity > 99.5%, IS) were purchased from the National Institutes for Food and Drug Control (Beijing, China).The Aβ_1–42_ peptide was provided by Sigma-Aldrich (St. Louis, MO, USA). HPLC-grade methanol was obtained from Fisher Scientific (Fair Lawn, NJ, USA). Ethanol, diethyl ether of analytical grade were purchased from Shandong Yuwang Industrial (Yucheng, China). Distilled water was provided by Wahaha Co. Ltd. (Hangzhou, China).

### 3.2. The Extract Preparation of Alpinia oxyphylla

The air-dried fruits of *A. oxyphylla* were extracted with 95% ethanol under reflux 3 times for 2 h each time. After being concentrated under vacuum, the extract was suspended in 0.5% CMC-Na for administration to the rats.

### 3.3. Animals and Treatment

Male SPF grade Sprague-Dawley (SD) rats (230 ± 20 g) were obtained from the Experimental Animal Center of Shenyang Pharmaceutical University. The animal study was performed according to the Guideline for Animal Experimentation of Shenyang Pharmaceutical University and the protocol was approved by the Animal Ethics Committee of the institution (SCXK 2015-001).

Twelve SD rats were divided into two groups randomly and given different treatments. The rats in the model group were anesthetized with chloralhydrate (200 mg/kg body weight, i.p.) and injected with aggregated 10 μL of the Aβ_1–42_ peptide (Aβ_1–42_ peptide was dissolved in physiological saline to a concentration of 1.0 μg/μL and incubated at 37 °C for 5 d to get the aggregated form) into the left lateral ventricle (AP, −3.0 mm, ML, ±2.2 mm, DV, −3.0 mm) [36].

### 3.4. The Morris Water Maze Test

To demonstrate the success of the model after Aβ injection, a water maze test was carried out by the Morris method with minor modification [37]. Parameters included escape latency time in order to find a platform, search strategy, and swimming time in the target quadrant, which were recoded for evaluating the spatial learning ability and memory of the tested object.

### 3.5. Instruments and UPLC–MS/MS Conditions

**Figure 6:**
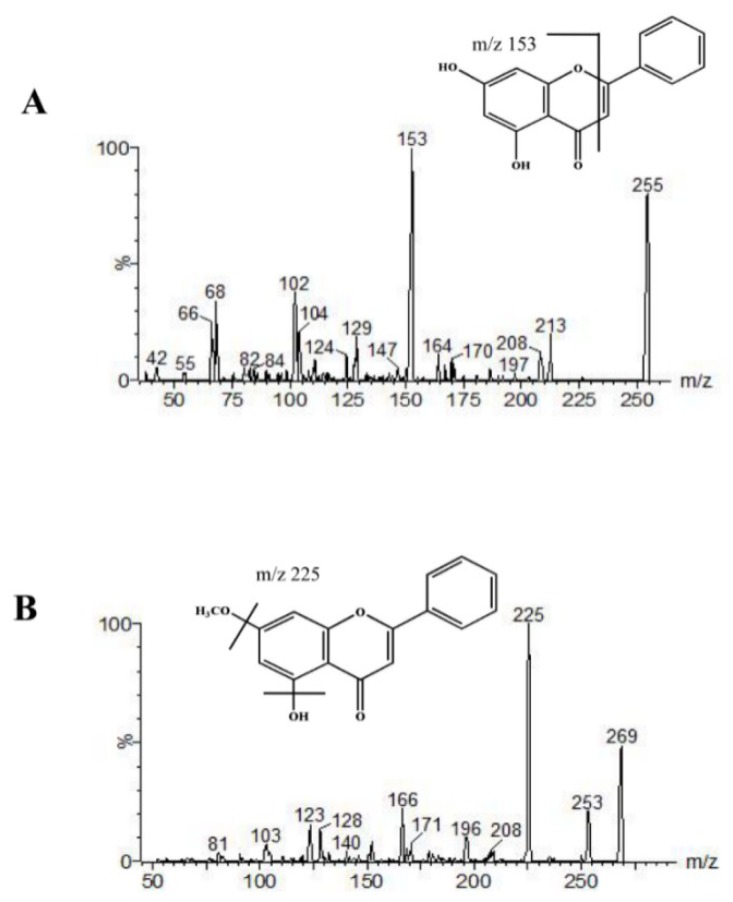
Product ion mass spectra of [M + H]^+^ ions of chrysin (**A**) and tectochrysin (**B**) in positive mode.

The assay was performed on an Acquity™ ultra-performance liquid chromatography system coupled with the Xevo TQ-S mass spectrometer (Waters, Milford, MA, USA). MassLynx software V4.1 was used for data acquisition and processing. The separation was conducted using an Acquity UPLC BEH C_18_ column (2.1 mm × 100 mm, 1.7 μm, Waters, Milford, MA, USA) packed with a BEH C_18_ pre-column (2.1 mm × 5 mm, 1.7 μm) with column temperature setting at 35 °C. Gradient elution was employed with a mobile phase composed of 0.1% formic acid-water (A) and methanol (B) with the following parameters: 0–2 min, 65–75% B, 2.01–3.5 min, 75–90% B, 3.51–5.0 min, 65% B. The mass spectrometer was operated in multiple reaction monitoring modes (MRM) using electrospray in the positive ionization mode with two precursor ion/product ion transitions for each analyte. The precursor-to-product ion pairs for chrysin, tectochrysin, and paclitaxel were *m/z* 255.0 → 153.1, 269.0 → 225.7, and 876.5 → 308.0, respectively (Figure 6). Other optimal parameters include a curtain voltage of 3.0 kV, a source temperature of 150 °C, and a desolvation temperature of 350 °C.

### 3.6. Standard Solution and Quality Control Samples

Standard stock solutions of chrysin, tectochrysin, and IS were prepared in methanol at concentrations of 1.0 mg/mL. Calibration standard solutions for chrysin and tectochrysin were prepared by diluting mixed stock solutions with methanol. IS solution was obtained by diluting to a concentration of 500 ng/mL with methanol as well.

Calibration standards of chrysin and tectochrysin (0.1 ng/mL, 0.25 ng/mL, 1.0 ng/mL, 5.0 ng/mL, 10 ng/mL, 25 ng/mL, and 50 ng/mL) were obtained by spiking a series of the working standard solution to drug-free rat plasma. Quality control (QC) samples at three levels (0.2 ng/mL, 2.0 ng/mL, and 40 ng/mL) were prepared separately in the same way.

### 3.7. Sample Preparation

Plasma samples of 100 μL were spiked with 10 μL methanol and 10 μL IS (500 ng/mL) and extracted with diethyl ether (1 mL) by vortex shaking for 5 min. The samples were centrifuged at 4000 rpm for 5 min and then the organic layer was transferred to another tube and evaporated to dryness at 35 °C under a slight stream of nitrogen. Then the residue was reconstituted with a 100 μL initial mobile phase and vortexing for 1 min. Finally, 5 μL of the solution was injected for UPLC–MS/MS analysis.

### 3.8. Method Validation

The method was validated in accordance with US FDA guidelines [38]. Selectivity was tested by comparing chromatograms of blank rat plasma with plasma samples spiked with two analytes and the IS at LLOQ. The linearity was measured by analyzing the calibration curves using least-squares linear regression analysis of the analytes-to-IS peak area ratios versus the nominal concentration of the calibration standard with a weighed factor (1/*x*^2^). LLOQ was defined as the lowest concentration on the calibration curve with an acceptable accuracy within ±20% and precision less than 20%. QC samples at low, medium, and high levels were analyzed on three separate occasions with six replicates at each concentration per occasion to determine accuracy and precision. The extraction recovery of the two analytes were assessed at three QC concentrations with six replicates by comparing the peak areas from extracted samples with those from post-extracted blank plasma samples spiked with the analytes. The matrix effect was determined by comparing the peak response of blank plasma extracts spiked with the analytes to the peak response of a pure standard solution containing equivalent amounts of the compounds at three QC levels. Stability studies in plasma samples were assessed at three QC levels under four different storage conditions, which include room temperature for 12 h, frozen at −20 °C for 30 d, three freeze-thaw cycles, and samples after being prepared at 4 °C for 12 h. The acceptable criteria of accuracy, extraction recovery, matrix effect, and stability were all within ±15% and the precision was less than 15%.

### 3.9. Pharmacokinetic Application

The method was applied toward determining chrysin and tectochrysin levels in rat plasma after oral administration of ethanol extract of *Alpinia oxyphylla* fruits (at a dose containing chrysin 0.25 mg/kg, tectochrysin 1.0 mg/kg). Blood samples (about 0.3 mL) were obtained from the suborbital vein into heparinized tubes before administration and 0.08 h, 0.17 h, 0.25 h, 0.5 h, 1 h, 2 h, 4 h, 6 h, 8 h, 12 h, and 24 h after dosing. It was then centrifuged at 15,000 rpm for 5 min immediately. Plasma samples were stored at −20 °C.

The plasma concentrations of two analytes at different points were presented as mean ± SD. The mean concentration-time curves were plotted and all the pharmacokinetic parameters were calculated using DAS 2.1 software package (Chinese Pharmacological Society, Beijing, China). Comparisons of pharmacokinetic data between normal and dementia groups were processed by using SPSS software 19.0 where *p* < 0.05 was considered statistically significant for all the tests.

## 4. Conclusions

A simple and sensitive UPLC–MS/MS method has been established and validated for determining chrysin and tectochrysin in rat plasma. The method has been successfully applied to a comparative pharmacokinetic study between normal rats and dementia rats after oral administration of the ethanol extract of *Alpinia oxyphylla* dried fruits. The results indicated that the absorption of the two compounds was significantly increased in dementia rats than that in normal rats. The established method and the pharmacokinetic results could provide useful information for preclinical studies of active constituents in *A. oxyphylla*, which has potential therapeutic effects on neurodegenerative diseases.

## Figures and Tables

**Table 1:** Accuracy, precision, extraction recovery, and matrix effect for analysis of chrysin and tectochrysin in rat plasma (*n* = 6).

	Spiked Concentration (ng/mL)	Accuracy (RE%)	Intra-Day RSD (%)	Inter-Day RSD (%)	Recovery (%, Mean ± SD)	Matrix Effect (%, Mean ± SD)
Chrysin	0.2	6.9	9.8	9.3	80.3 ± 3.7	96.9 ± 2.9
	2.0	7.5	5.6	6.2	84.6 ± 5.5	97.3 ± 3.8
	40	−8.8	4.9	5.0	86.7 ± 2.8	97.8 ± 3.1
Tectochrysin	0.2	5.6	7.4	8.7	81.5 ± 4.1	98.2 ± 4.5
	2.0	4.4	3.7	6.4	85.2 ± 3.9	97.7 ± 4.2
	40	−6.8	2.6	3.8	85.0 ± 3.5	97.1 ± 3.6

**Table 2:** Stability of chrysin and tectochrysin in rat plasma (*n* = 3).

Conditions	Chrysin			Tectochrysin		
	Spiked (ng/mL)	RE (%)	RSD (%)	Spiked (ng/mL)	RE (%)	RSD (%)
Room temperature for 12 h	0.2	4.9	7.8	0.2	5.8	7.2
	2.0	−2.7	5.6	2.0	2.3	6.5
	40	1.6	4.1	40	3.1	5.6
Frozen for 30 d	0.2	−2.6	8.6	0.2	−4.9	8.4
	2.0	1.3	4.4	2.0	−3.7	4.6
	40	3.8	6.2	40	2.5	5.1
Three freeze and thaw cycles	0.2	−2.5	9.9	0.2	−1.1	6.5
	2.0	−5.6	4.6	2.0	−2.9	5.7
	40	2.2	3.8	40	−0.4	4.4
4 °C in auto-sampler for 12 h in processed samples	0.2	0.8	9.5	0.2	0.6	7.4
	2.0	4.4	4.7	2.0	3.6	6.8
	40	2.4	4.0	40	2.5	5.3

**Table 3:** Main pharmacokinetic parameters of chrysin and tectochrysin after oral administration of *Alpinia oxyphylla* fruit extract (mean ± SD, *n* = 6).

Parameters (Unit)	Chrysin		Tectochrysin	
	Normal Rat	Dementia Rat	Normal Rat	Dementia Rat
AUC_0–t_ (ng h/mL)	78.81 ± 25.21	131.1 ± 44.6 **	10.99 ± 3.45	20.40 ± 4.54 **
AUC_0–∞_ (ng h/mL)	81.6 ± 29.34	135.1 ± 47.4 **	13.25 ± 3.98	23.59 ± 4.68 **
*C*_max_ (ng/mL)	18.06 ± 4.31	29.28 ± 8.72 *	9.87 ± 3.01	19.29 ± 5.05 **
*t*_1/2_ (h)	4.51 ± 1.02	4.22 ± 0.89	3.72 ± 0.82	3.14 ± 0.71
*T*_max_ (h)	0.25, 8	0.25, 8	0.25	0.25
MRT (h)	7.52 ± 1.34	7.45 ± 1.19	4.12 ± 1.16	3.54 ± 1.11
CL_F (L·kg/h)	6.127 ± 1.514	3.702 ± 0.775 **	75.49 ± 30.85	42.39 ± 26.76 **

* *p* < 0.05, ** *p* < 0.01 compared with the normal rats.
